# Hydrogen sulfide alleviates neural degeneration probably by reducing oxidative stress and aldose reductase expression

**DOI:** 10.1111/jcmm.70192

**Published:** 2024-11-08

**Authors:** Wenqi Shen, Tingyu Hu, Xin Wang, Xiaoyan Zhang, Junxi Lu, Huijuan Lu, Yanyun Hu, Fang Liu

**Affiliations:** ^1^ Department of Endocrinology and Metabolism, Shanghai Sixth People’s Hospital Shanghai Jiaotong University Shanghai China; ^2^ Shanghai Jiaotong University School of Medicine Shanghai China; ^3^ Department of Endocrinology and Metabolism, Shanghai General Hospital Shanghai Jiaotong University School of Medicine Shanghai China

**Keywords:** aldose reductase, diabetic peripheral neuropathy, hydrogen sulfide, oxidative stress

## Abstract

We investigated the potential role of hydrogen sulfide (H_2_S) as a novel therapy for diabetic peripheral neuropathy in diabetic rats. A single dose of streptozotocin (60 mg/kg) was applied to the rats for the diabetic rat models. Sodium bisulfide (50 μmol/kg/d) was injected intraperitoneally daily for 2 weeks as H_2_S treatment. Electromyogram, haematoxylin eosin staining, transmission electron microscopy, western blotting and enzyme‐linked immunosorbent assay were then performed. H_2_S treatment did not affect body weights, blood glucose levels or liver function of diabetic rats, while the creatine levels of the H_2_S‐treated diabetic rats decreased compared with the diabetic control rats. H_2_S treatment for 2 weeks did not affect the sciatic nerve conduction velocity of the diabetic rats. However, H_2_S treatment relieved neurons loss and cell atrophy of dorsal root ganglion, and axon degeneration of sciatic nerve in diabetic rats. Serum super oxide dismutase (SOD) levels and SOD2 levels in the sciatic nerve of diabetic rats were lower than the non‐diabetic rats but were restored after H_2_S treatment. Serum and sciatic nerve homogenate malondialdehyde and aldose reductase expression were higher in diabetic rats but decreased significantly after H_2_S treatment. Our study revealed that H_2_S alleviates neural degeneration in diabetic rats probably by reducing oxidative stress and downregulating aldose reductase expression.

## 1 INTRODUCTION

Diabetic peripheral neuropathy (DPN) is one of the most prevalent long‐term complications of diabetes, affecting up to 50% of diabetes patients.^1^ Distal symmetrical sensory multiple neuropathy is the most prevalent type of DPN. It usually leads to limb pain, numbness and abnormal activity. DPN diminishes the quality of life and contributes significantly to the development of diabetic foot. Globally, DPN is the leading cause of non‐traumatic amputations.^2^, ^3^ Therefore, it places a heavy burden on both families and the society.

Extensive efforts have been made to explore the pathogenesis of DPN. Oxidative stress, inflammation, micro‐vascular lesions, polyol pathway activation and deficits in neurotrophic factors caused by long‐term hyperglycemia may be involved in the onset and development of DPN.^4^ Existing DPN therapies, including various medicines (mecobalamine, zincalpha sulfate, epalrestat, etc.) and physiotherapies (spinal cord electrical stimulation, etc.) are not satisfactory.^5^ Hence, there is a need for novel therapies to treat DPN.

In 1996, Abe and Kimura introduced hydrogen sulfide (H_2_S) as an endogenous neuro‐regulatory substance.^6^ Subsequent research showed that H_2_S is involved in a variety of physiological functions across the respiratory, cardiovascular and neural systems.^7^ Currently, H_2_S is recognized as the third class of endogenous gas signalling molecules along with nitric oxide and carbon monoxide.^8^ It is related to a variety of physiological and pathological processes in the human body, including anti‐oxidative stress, anti‐inflammatory responses, promotion of angiogenesis and interference with apoptosis.^9^ H_2_S exerts its antioxidant effect by limiting free radical reactions through the activation of antioxidant enzymes, including super oxide dismutase (SOD), catalase and glutathione peroxidas.^10^ And it protects against aging by regulating apoptosis‐related genes, including p53, B‐cell lymphoma (Bcl) associated X protein and Bcl‐2.^11^ H_2_S also alleviates age‐associated neurodegenerative diseases, including Alzheimer's disease, Parkinson's disease, Huntington's disease and Down's syndrome.^12^, ^13^, ^14^, ^15^ However, so far, the effect of H_2_S on DPN is yet to be studied extensively. Therefore, it is important to investigate how H_2_S can potentially affect diabetic neuropathy and understand the underlying mechanisms, which might lead to novel and effective treatments for DPN.

## 2 MATERIALS AND METHODS

### 2.1 Materials

Streptozotocin (STZ), citric acid, sodium citrate and sodium hydrosulfide (NaHS) were purchased from Sigma Aldrich, USA. The SOD kit was purchased from DOJINDO Laboratory, Japan. The Malondialdehyde (MDA) assay kit was purchased from Nanjing Jiancheng Bioengineering Research Institute. The primary antibodies including anti‐SOD2 (1:5000, ab13533) and anti‐aldose reductase (AR) (1:2000, ab175394) were purchased from Abcam. The secondary antibody anti‐Immunoglobin was purchased from Cell Signalling Technology (1:1000, 7074).

### 2.2 Animal model of diabetic peripheral neuropathy

All animal experiments were performed in accordance with the China Council for Animal Care and Use guidelines. This study was approved by the Ethical Committee of Experimental Animals of Shanghai Sixth People's Hospital (2019–53‐(2)). The study was performed in accordance with the Declaration of Helsinki. Thirty specific‐pathogen‐free Sprague–Dawley rats of 8‐weeks‐old, weighing 190–210 g were purchased from the Model Animal Research Center of Nanjing University. The animals were allowed to acclimatize to the laboratory conditions for 1 week. After 8 h of fasting, a single dose of intraperitoneal STZ injection (60 mg/kg) in sterile citrate buffer (0.1 M; pH 4.2–4.4) was injected intraperitoneally to establish the diabetes model (*n* = 20). Equal volumes of citrate buffer were injected intraperitoneally into the non‐diabetic control rats (NC, *n* = 10). Fasting blood samples were collected 3 days after STZ administration to measure blood glucose using a glucometer (Roche, Germany). Rats with fasting glucose levels over 16.6 mmol/L were considered to have diabetes. DPN models were established 6 weeks after STZ administration.^16^ Throughout the experiment, the body weights and fasting blood glucose levels of the rats were measured every 2 weeks. None of the rats died or experienced serious adverse events during the experiment.

### 2.3 H_2_S treatment

The diabetic rats were then randomly divided into the diabetic control group (*n* = 10) and the H₂S‐treated diabetic group (*n* = 10). Following our previous study, we used NaHS solution as the H_2_S donor.^17^ NaHS is a simple and effective H₂S donor that releases H₂S immediately when exposed to water, therefore it is commonly used in biochemical and physiological studies.^18^ 10.5 mg of NaHS was weighed and added to 18.75 mL of saline to make a 10 mM NaHS solution, which was administered to the rats by intraperitoneal injection at a dose of 50 μmol/(kg/d). Based on previous studies, the dosage used is not toxic to the rats.^19^ Six weeks after the STZ injection, NaHS was administered daily for 2 weeks in the H_2_S‐treated diabetic group. And the same volume of saline was administrated daily to the non‐diabetic control group and the diabetic control group for 2 weeks.

### 2.4 Measurement of the sciatic nerve's motor nerve conduction velocity

After the final NaHS administration, the animals were anaesthetised intraperitoneally with 0.6% sodium pentobarbital solution (9 mL/kg) intraperitoneally, and the sciatic nerve was then exposed. Motor nerve conduction velocity (MNCV) (m/s) was measured by one professional technician with a Power Lab 8S system (Denmark). One electrode was placed on the sciatic nerve near the obturator foramen and the other on the common peroneal nerve just distal to the division. Each nerve conduction velocity was measured three times and the average values were then recorded. MNCV was defined as the distance between the distal and proximal stimulation electrodes divided by the difference in latency.

### 2.5 Tissue extraction and biochemical investigations

After the rats were anaesthetised, we collected the sciatic nerve and dorsal root ganglion (DRG). Blood samples were taken from the abdominal aorta and spun for 10 min at 2683 g and then frozen at −80°C if the biochemical analysis could not be performed immediately. Serum triglyceride (TG), total cholesterol (TC), low‐density lipoprotein cholesterol (LDL‐c), high‐density lipoprotein cholesterol (HDL‐c), blood urea nitrogen (BUN), creatinine (Cr), alanine aminotransferase (ALT) and aspartate acid transaminase (AST) levels were tested by an automatic biochemistry analyser (Rayto Technologies, China).

### 2.6 Light microscopy

Immediately after collection, the sciatic nerve and DRG were fixed in 4% paraformaldehyde solution for 24 h and rinsed fully under running water. The tissues were soaked sequentially in 75%, 90%, 95% and 100% ethanol for dehydration. Then they were immersed in xylene and immersed in paraffin wax at 60–65°C for embedding. The embedded paraffin blocks were serially sectioned at a thickness of 4 μm. Sections were processed and stained with haematoxylin and eosin (HE) according to a standard procedure, and examined under a light microscope (Leica Microsystems, Germany).

### 2.7 Transmission electron microscopy observations

Nerve tissues were cut into small pieces and then fixed in 2.5% glutaraldehyde, post‐fixed in 1% osmium tetroxide, dehydrated in an ascending series of alcohols and finally embedded in epoxy resin. The ultrathin sections stained with uranyl acetate and lead citrate were examined using a PHILIPS CM‐120 transmission electron microscope (FEI, Eindhoven, Netherlands). Examiners blinded to the experimental design performed the morphometric evaluations. At least three random high‐power fields were selected for observation and calculation. Image‐Pro Plus (Media Cybernetics Inc., USA) was used for data processing, including axon diameter, fibre diameter, G‐ratio (the ratio of the inner to the outer diameter of the myelin sheath of a myelinated axon), integrated optical density, myelin thickness and number of nerve fibres.

### 2.8 Western blotting

Briefly, sciatic nerves were harvested and lysed in pre‐chilled radioimmunoprecipitation assay buffer containing a phosphatase inhibitor cocktail and phenyl methyl sulfonyl fluoride for 10 min on ice. The lysates were diluted with 5× loading buffer. Protein extracts were boiled at 95°C for 5 min. The proteins were separated using 10–20% gradient sodium dodecyl sulfate‐polyacrylamide gel electrophoresis (SDS‐PAGE) (Bio‐Rad Laboratories, United States). The extracted proteins were transferred from SDS‐PAGE (Bio‐Rad Laboratories, Hercules, CA, USA) onto a polyvinylidene fluoride membrane (Millipore Corporation). The membranes were blocked with 5% skimmed milk and incubated with primary antibodies overnight and then incubated with secondary antibodies for 1 h. The protein bands on the membrane were visualized using an enhanced chemiluminescence substrate kit (Thermo Fisher Scientific Inc., USA). The band intensities were quantitated using ImageJ software.

### 2.9 Enzyme‐linked immunosorbent assay (ELISA) for detecting serum MDA and SOD

Serum and sciatic nerve homogenate MDA levels were measured using the MDA kit from Nanjing Jiancheng Bioengineering Research Institute following the manufacturer's specifications. SOD levels were measured using a SOD kit from DOJINDO Laboratory following the manufacturer's protocols.

### 2.10 Statistical analysis

Data were collected and analysed using the statistical package for social sciences (version 25.0; SPSS, Chicago, IL, USA) and Excel (Microsoft, Redmond, WA, USA). Charts were prepared using GraphPad Prism8. Quantitative data conforming to normal distribution were expressed as mean ± SD. Comparisons between groups were performed by a one‐way analysis of variance (ANOVA). Pairwise comparisons between the groups were performed with the Least‐Significant Difference (LSD) test. Two‐sided *p* < 0.05 was considered statistically significant.

## 3 RESULTS

### 3.1 H_2_S treatment did not affect the liver function and lipid profiles of the rats

We measured serum ALT, AST, BUN, Cr, TG, TC, HDL‐c and LDL‐c levels in each group to determine whether H_2_S treatment affects liver/kidney function and lipid profiles. Compared to the non‐diabetic control group, the ALT, BUN, TC and HDL‐c levels were all increased in both the diabetic control group and the H_2_S‐treated diabetic group (*p* < 0.05). The parameters were not significantly different between the diabetic control and the H_2_S‐treated groups, except for the serum Cr level, which was lower in the H_2_S‐reated group than in the diabetic control group (*p* < 0.05) (Table 1).

**TABLE 1:** Comparison of liver/kidney function and lipid profiles in the non‐diabetic control group (NC), diabetic control group (DM) and H_2_S‐treated group (H_2_S). n=10 for each group

	NC	DM	H_2_S	*p*
ALT, μ/L	46.72 ± 8.24	84.41 ± 18.77*	76.88 ± 21.35*	0.001
AST, μ/L	167.99 ± 57.56	168.67 ± 54.96	169.65 ± 34.11	0.980
BUN, mg/dl	13.12 ± 3.33	19.53 ± 3.60*	18.50 ± 2.25*	0.001
Cr, μmol/L	33.61 ± 6.76	46.14 ± 9.53*	33.22 ± 11.84^#^	0.047
TG, mmol/L	1.78 ± 0.45	2.08 ± 1.08	1.68 ± 0.24	0.501
TC, mmol/L	2.44 ± 0.27	3.17 ± 0.65*	2.86 ± 0.43	0.008
HDL‐c, mmol/L	0.28 ± 0.07	0.42 ± 0.14*	0.39 ± 0.10*	0.119
LDL‐c, mmol/L	1.50 ± 0.16	1.92 ± 0.42*	1.70 ± 0.24	0.075

*Note*: Continuous data are presented as mean ± SD.

Abbreviations: ALT, Alanine aminotransferase; AST, Aspartic acid transaminase; BUN, Blood urea nitrogen; Cr, Creatinine; HDL‐c, High‐density lipoprotein cholesterol; LDL‐c, Low‐density lipoprotein cholesterol; TC, Total cholesterol; TG, Triglyceride.

* Indicates *p* < 0.05 vs. NC.

^#^ Indicates *p* < 0.05 vs. DM.

### 3.2 H_2_S treatment did not affect the plasma glucose levels or weights of diabetic rats

**FIGURE 1:**
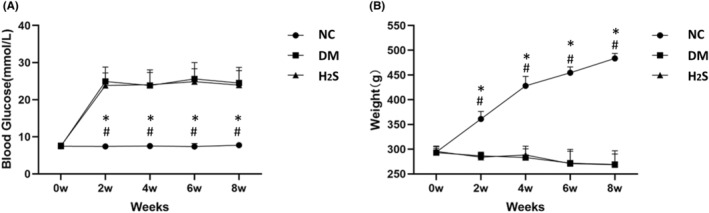
H_2_S treatment did not affect the plasma glucose levels or weights of diabetic rats. (A). Fasting blood glucose levels of rats over time. (B). Weights of rats over time. * indicates *p* < 0.05 vs. diabetic group (DM), # indicates *p* < 0.05 vs. H_2_S‐treated group (H_2_S). NC indicates the non‐diabetic control group. *n* = 10 for each group.

Figure 1 shows the blood glucose levels and body weights of the rats. The glucose levels of the diabetic control group and the H_2_S‐treated diabetic group were significantly higher than the non‐diabetic control group (*p* < 0.01), and there was no difference between the diabetic control group and the H_2_S‐treated diabetic group (*p* > 0.05). The body weights of the non‐diabetic control group increased gradually over time, whereas they slightly decreased without an intergroup difference in the diabetic control group and the H_2_S‐treated diabetic group (*p* > 0.05) (Figure 1).

### 3.3 H_2_S treatment for 2 weeks did not affect MNCV of diabetic rats

**FIGURE 2:**
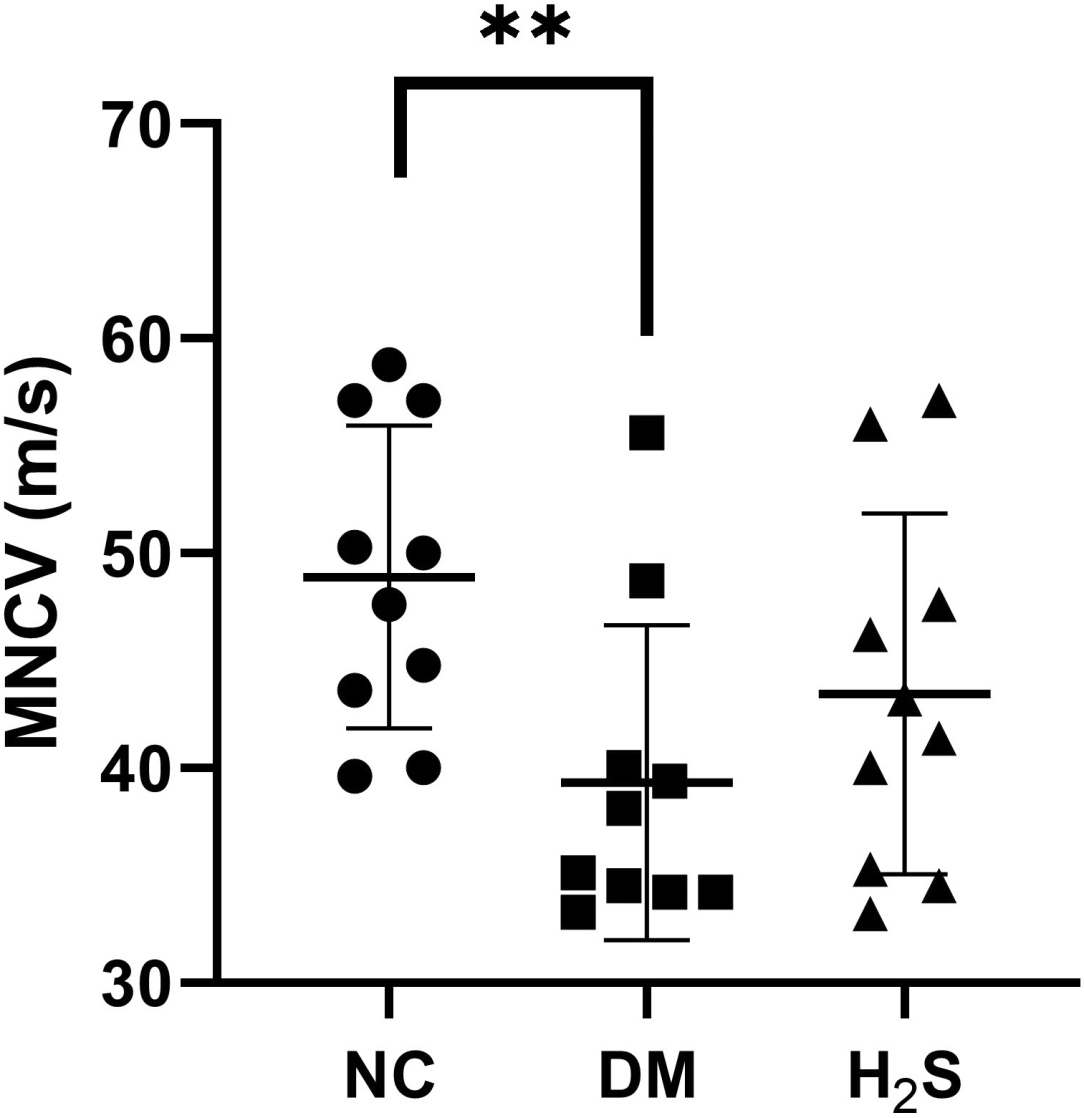
H_2_S treatment did not affect the motor nerve conduction velocity (MNCV) of diabetic rats. Comparison of MNCV in three groups. ** indicates *p* < 0.01 non‐diabetic control group (NC) vs. diabetic control group (DM). No significant difference between the H_2_S‐treated diabetic group (H_2_S) and DM group (*p* = 0.076). *n* = 10 for each group.

We tested the MNCV of diabetic and non‐diabetic rats. The sciatic nerve MNCV of the diabetic control rats was much slower than the non‐diabetic control group (39.31 ± 7.34 m/s vs. 48.89 ± 7.06 m/s, *p* < 0.01), while there was no statistical significance between the diabetic control group and the H_2_S‐treated diabetic group after H_2_S treatment for 2 weeks (43.45 ± 8.41 m/s vs. 39.31 ± 7.34 m/s, *p* = 0.076) (Figure 2).

### 3.4 H_2_S treatment reduced neural degeneration

**FIGURE 3:**
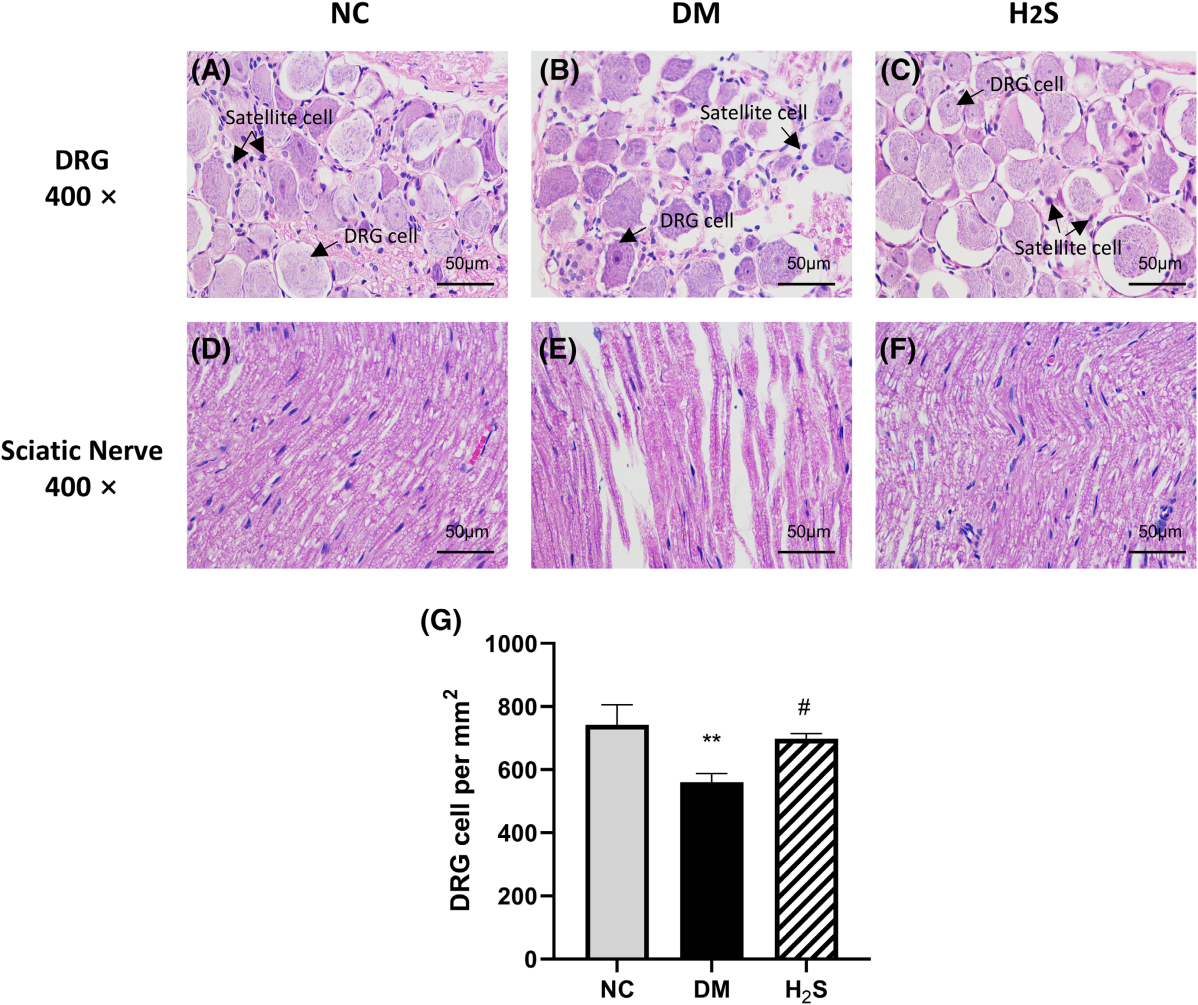
H_2_S treatment reduced neural degeneration in diabetic rats. Histological examination of DRG and sciatic nerve with HE staining at magnification 400x, scale bar: 50um. (A). Representative DRG tissue section of non‐diabetic control group (NC) demonstrates healthy neurons surrounded by a thin layer of supportive satellite glial cells. (B). In diabetic rats (DM) DRG neurons were sparser, neurons shrank and exposed a stronger basophilic staining attitude. Multilayered satellite glial cells could be observed around the DRG neurons. (C). After H_2_S treatment, DRG neurons density increased and all the pathological changes in DM were relieved. (D). Normal myelinated fibres of sciatic nerves were arranged densely and wrapped with myelin. (E). Sciatic nerves of DM group exhibited nerve fibre loss and demyelination changes. (F)Nerve fibre loss and demyelination changes were restored after H_2_S treatment. (G). Comparison of quantification of DRG density among three groups. ** indicates *p* < 0.01 vs. non‐diabetic control group (NC), # indicates *p* < 0.05 vs. diabetic group (DM). *n* = 10 for each group.

We performed HE staining and transmission electron microscopy to observe the morphological change of DRG and the sciatic nerve. HE staining revealed normal DRG and sciatic nerve structure in the non‐diabetic control group. In the diabetic control group, there was an overall decrease in the DRG neurons number (*p* < 0.01) and cell body atrophy could be observed, the DRG neurons exposed a stronger basophilic staining attitude. Besides, the number of satellite glial cells around the DRG neurons increased and multilayered satellite glial cells could be observed. After H_2_S treatment, DRG neurons number increased (*p* < 0.05) and all the pathological changes in DM were relieved. (Figure 3). In the sciatic nerve sections of the diabetic control group, the number of nerve fibres were decreased and myelin sheath deteriorated. Nerve fibre loss and demyelination changes were restored after H_2_S treatment. (Figure 3).

**FIGURE 4:**
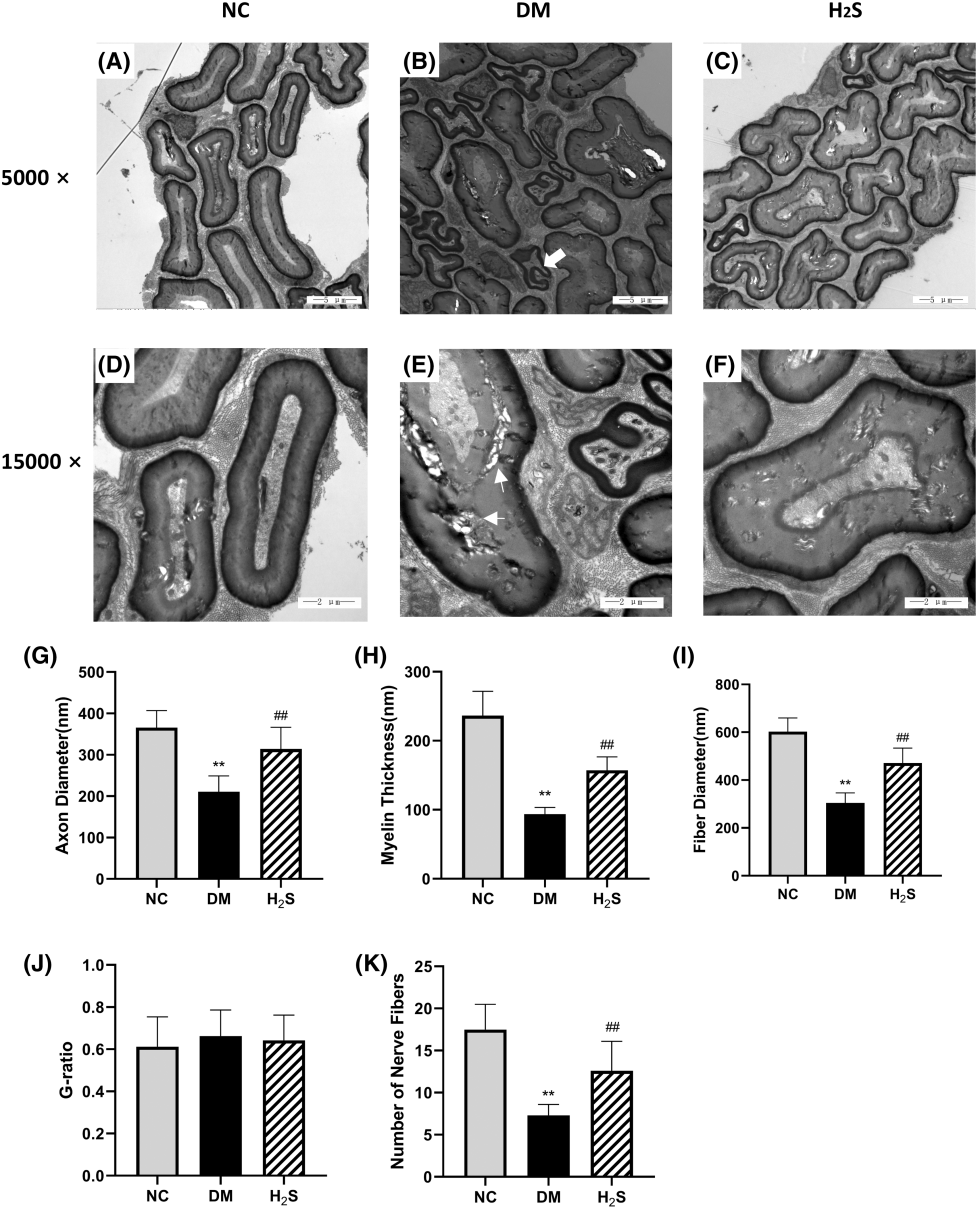
H_2_S treatment alleviated the axon degeneration of the sciatic nerve in diabetic rats. (A). Representative electron microscopy images of the sciatic nerve at magnifications 5000x and 15,000x, scale bar: 2 μm. (A, D). The morphology of the sciatic nerve was normal, the myelin sheaths arranged densely and there was no obvious axonal degeneration and myelin loss. (B, E). The normal morphology of the sciatic nerve disappeared, axon degeneration (variable thickness of myelinated nerve fibres, enfolding and out folding nerve fibres (thick arrow) and vacuolization of the myelin sheath (thin arrow)) could be observed. (C, F). Axon degeneration was partly relieved after H_2_S treatment. Quantitative comparison of (G)axon diameter (nm), (H)myelin thickness (nm), (I)fibre diameter (nm), (J) G‐ratio, (K)number of nerve fibres of the sciatic nerve. ** indicates *p* < 0.01 vs. non‐diabetic control group (NC), ## indicates *p* < 0.01 vs. diabetic group (DM). ‘H_2_S’ indicates the H_2_S treated group. *n* = 10 for each group.

Transmission electron microscopy revealed normal axons with uniform and dense myelination and structural integrity in the sciatic nerve of non‐diabetic rats (Figure 4A,D). In the diabetic control group, the normal morphology of the sciatic nerve disappeared, axon degeneration (variable thickness of myelinated nerve fibres, enfolding and out folding nerve fibres and vacuolization of the myelin sheath) could be observed (Figure 4B,E). After H_2_S treatment, axon degeneration was partly relieved (Figure 4C,F), and the axon diameter, myelin thickness and fibre length increased (*p* < 0.01) (Figure 4G–I). The number of nerve fibres also increased (*p* < 0.01) (Figure 4K). However, no significant differences were found in the G‐ratios among the three groups (*p* > 0.05) (Figure 4J).

### 3.5 H_2_S treatment reduced oxidative stress and downregulated the AR level in sciatic nerve

**FIGURE 5:**
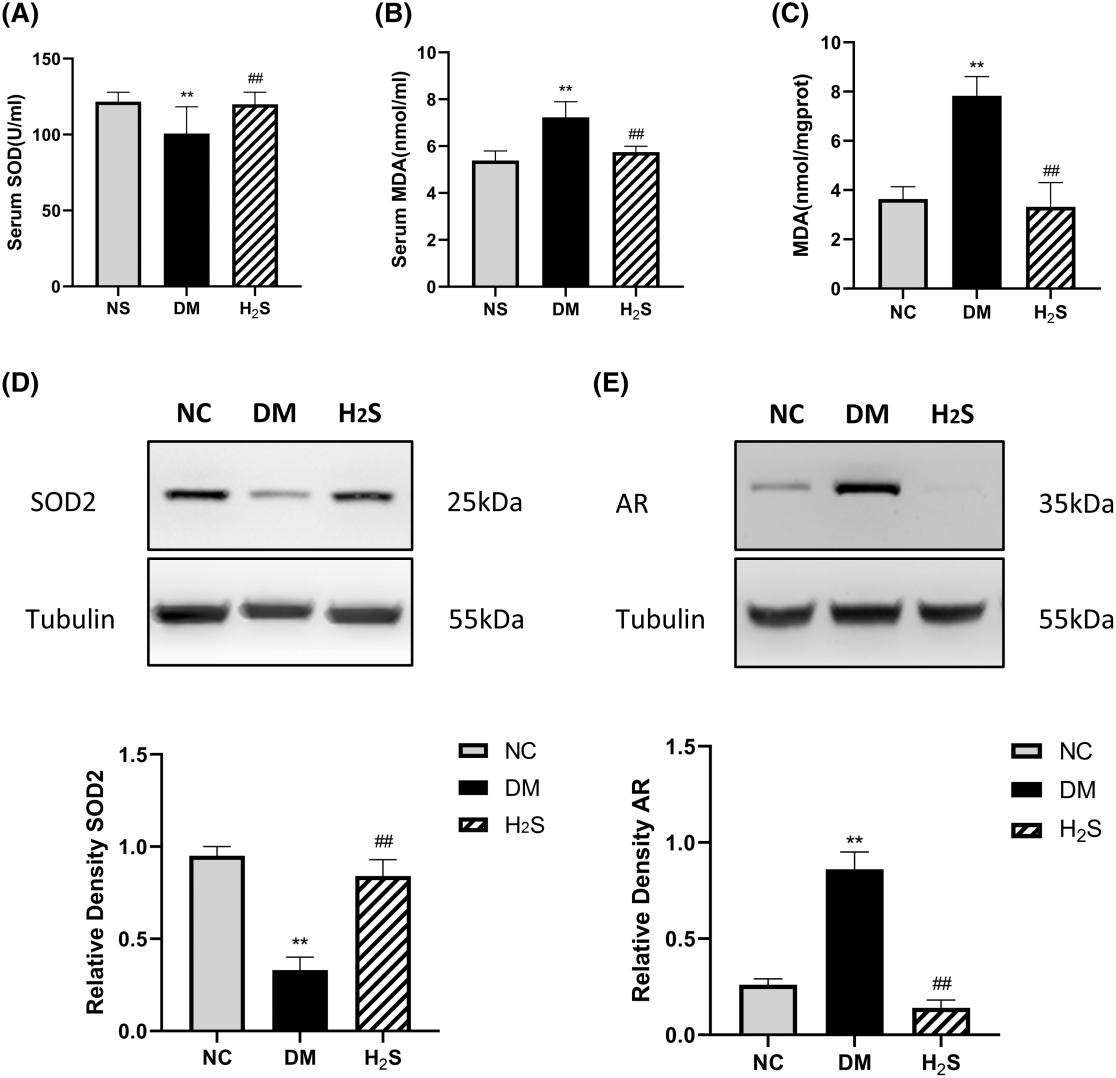
H_2_S treatment reduced oxidative stress and down‐regulates the AR level in diabetic rats. Comparison of (A) serum SOD, (B) serum MDA, (C) MDA in sciatic nerve, (D) SOD2 in sciatic nerve, (E) AR in sciatic nerve. ** indicates *p* < 0.01 vs. non‐diabetic control group (NC), ## indicates *p* < 0.01 vs. diabetic group (DM). ‘H_2_S’ indicates the H_2_S treated group. *n* = 10 for each group.

Oxidative stress plays an important role in DPN.^20^ In our study, the serum SOD levels of the diabetic control rats were much lower than the non‐diabetic control rats (*p* < 0.01). After H_2_S treatment, the serum SOD levels increased significantly (*p* < 0.01) (Figure 5A). We also tested the SOD2 levels in the sciatic nerve using western blot (Figure 5D). The diabetic control group had lower SOD2 levels compared with the non‐diabetic control group, which were restored after H_2_S treatment (Figure 5D). Serum and sciatic nerve MDA levels and were both significantly higher in the diabetic control group (*p* < 0.01), and both decreased after H_2_S treatment (*p* < 0.01) (Figure 5B,C).

Over‐activation of the Polyol Pathway is critical in diabetic peripheral neuropathy.^21^ The AR level in the sciatic nerve was determined by western blotting. AR expression increased significantly in the diabetic control group and decreased after H_2_S treatment (*p* < 0.01) (Figure 5E).

## 4 DISCUSSION

In this study, we observed that H_2_S attenuated nerve fibre loss and axon degeneration, which are typical nerve injury manifestations in DPN.^22^ However, H_2_S treatment for 2 weeks did not affect MNCV of diabetic rats. In addition, serum SOD and sciatic nerve SOD2 levels of diabetic rats both increased, while MDA levels in both serum and sciatic nerve decreased after H_2_S treatment. AR expression also decreased after H_2_S treatment in diabetic rats. These findings indicate that H_2_S may attenuate neural degeneration in DPN by reducing oxidative stress and downregulating aldose reductase expression.

Former studies have proved that different H_2_S donors are safe for animals.^14^, ^16^, ^17^ A study from Hanan et al. has proved that NaHS reversed biochemical, apoptotic, oxidant and pathologic parameters characteristic of diabetic nephropathy.^23^ Another novel hydrogen sulfide‐releasing compound, S‐propargyl‐cysteine treatment significantly reduced the level of creatinine, kidney to body weight ratio and in particular markedly decreased 24‐h urine microalbuminuria excretion.^24^ In this study, we also observed that the serum Cr levels of the diabetic rats decreased after H_2_S treatment, indicating that H_2_S treatment relieved diabetic nephropathy.

Previous studies have demonstrated that endogenous H_2_S can alleviate age‐associated neurodegenerative diseases.^12^ In Alzheimer's disease, H_2_S was diminished, while administering the H_2_S donor sodium GYY4137 ameliorated motor and cognitive deficits of 3xTg‐Alzheimer's disease mice.^14^ In mice with neurodegeneration and neurovascular dysfunction induced by intracerebral‐administered homocysteine, NaHS administration attenuated homocysteine‐induced oxidative stress, memory deficits, neurodegeneration, neuroinflammation and cerebrovascular remodelling.^15^ In animal models of neuropathic pain induced by chronic constriction injury of the sciatic nerve, inhaled H_2_S prevented the neuropathic pain behaviour, including mechanical allodynia and thermal hyperalgesia, and also attenuated the upregulation of the inflammatory cytokines interleukin 6 and chemokine CC motif ligand 2. A few studies have explored the role of H_2_S in diabetic and non‐diabetic pain. A study from M E Velasco‐Xolalpa et al. showed that H_2_S is involved in formalin‐induced nociception in diabetic and non‐diabetic rats, as well as tactile allodynia in STZ induced diabetic rats.^25^ Another study by Hao Li et al. proved that H_2_S might attenuate diabetic neuropathic pain through nitric oxide/cyclic guanosine monophosphate/protein kinase G pathway and μ‐opioid receptor.^26^ However, the role of H_2_S in DPN has not been extensively. Although the pathogenesis of DPN remains unclear, researchers have demonstrated that nerve fibre loss, paranodal and demyelination remyelination, segmental demyelination and Wallerian degeneration are major pathological alterations in rats and patients with diabetes.^27^, ^28^ In our study, neurocyte atrophy and axon degeneration characterized by nerve fibre loss, cell body atrophy and deteriorated myelin sheath in diabetic rats, were all alleviated after H_2_S treatment. These findings indicated that H_2_S relieved the neural degeneration in DPN rats.

Animal models of diabetes often exhibit functional and structural characteristics of neuropathy that resemble those observed in patients, including reduced conduction velocity of large fibres and diminished presence of small sensory fibres in the skin and cornea.^29^, ^30^, ^31^ In our study, the sciatic nerve MNCV decreased significantly in the diabetic control group compared with the non‐diabetic control group. However, there was no significant difference in sciatic nerve MNCV between the diabetic control group and the H_2_S‐treated group. The MNCV values of H_2_S treated diabetic rats varied probably due to the small sample size. And the H_2_S treatment only lasted for 2 weeks, which might not be long enough to restore the decrease in MNCV. The gradation of DPN in diabetic rats and the different responses to H_2_S treatment may also contribute to the diversion of MNCV values. In future studies, a larger sample size and prolonged treatment with NaHS may be helpful for further exploring how H_2_S treatment affects MNCV.

Metabolic disturbances, oxidative stress, endothelial dysfunction, dysregulation of neurotrophic factors, the polyol pathway activation and accumulation of advanced glycosylation end products (AGEs) are associated with DPN.^32^, ^33^, ^34^ Overproduction of reactive oxygen species (ROS) in the mitochondria and cytoplasm, combined with the deregulation of antioxidant defence systems, causes oxidative stress.^35^ Furthermore, oxidative stress plays a crucial role in the pathophysiology of DPN by interfering with other related pathways.^36^ SOD is a natural superoxide‐free radical scavenging factor in the body, which is the first line of defence against oxygen‐free radicals. MDA is a lipid peroxide produced by ROS in cell membranes. It is well known that the levels of MDA, the activity of SOD are the essential indicators of oxidative stress. The SOD2 levels slightly decreased in animals with DPN and the MDA levels increased obviously in animals with DPN.^37^ In our study, we tested the SOD and MDA levels in the serum and also SOD2 and the MDA levels in the sciatic nerves. A markable increase in MDA levels and a decrease in SOD/SOD2 were observed in the diabetic control group. And in the rats with DPN, MDA levels decreased, while SOD/SOD2activity increased after H_2_S treatment. These findings indicate that H_2_S can reduce nerve tissue oxidative stress in DPN nerve tissues.

Over‐activation of the polyol pathway is another factor contributing to DPN.^28^ A rise in the intracellular sorbitol concentration in diabetes causes osmotic stress, facilitates electrolyte efflux and affects peripheral Schwann cells. It has been demonstrated that the polyol pathway may contribute to the schwannopathy‐related phenotype of DPN.^38^ In addition, sorbitol and fructose accumulation reduces myoinositol and taurine concentrations, axon swelling, axon‐glia dysfunction and nerve conduction velocity reduction.^39^ AR and sorbitol dehydrogenase are critical enzymes involved in glucose metabolism through the polyol pathway. AR plays a pivotal role in the diabetes‐mediated up‐regulation of MAPK (mitogen‐activated protein kinase) activity in the nerve, spinal cord and dorsal root ganglia.^40^ Furthermore, we also observed that AR expression in the sciatic nerve was significantly higher in the diabetic control group but decreased after H_2_S treatment. These results indicated that H_2_S treatment may relieve DPN by inhibiting AR expression.

This study had some limitations. First, we did not assess the nociceptive functions, such as mechanical and thermal pain responses in the rats with diabetes. Assessing nociceptive functions could provide a more comprehensive understanding of the effects of H_2_S treatment on neuropathy. Secondly, it would be more convincing to detect ROS levels and AR activity to elucidate how H_2_S treatment affects the oxidative stress.

In conclusion, we preliminarily verified the protective effect of H_2_S on the neural degeneration in STZ‐induced diabetic rats with DPN and the safety of its application in animals. The mechanisms underlying the protective effects of H_2_S may be related to inhibiting oxidative stress and the polyol pathway. This may provide a novel therapeutic approach for DPN in the future.

## AUTHOR CONTRIBUTIONS

**Wenqi Shen:** Formal analysis (equal); investigation (lead); methodology (lead); writing – review and editing (equal). **Tingyu Hu:** Methodology (equal). **Xin Wang:** Methodology (equal). **Xiaoyan Zhang:** Methodology (equal); project administration (equal); writing – review and editing (equal). **Junxi Lu:** Data curation (equal); resources (equal); software (equal). **Huijuan Lu:** Data curation (equal); supervision (equal). **Yanyun Hu:** Data curation (equal); methodology (equal); writing – original draft (lead). **Fang Liu:** Funding acquisition (lead); project administration (lead); resources (lead); supervision (lead); writing – review and editing (equal).

## FUNDING INFORMATION

This research was supported by grants from the National Natural Science Found of China (82170827), Shanghai Natural Science Found of Shanghai Science and Technology Committee(22ZR1450100), Yangtze River Delta Field Regional Cooperation Projects(22002400600), Characteristic Talents Cultivation Program of Shanghai General Hospital (Shanghai Leading Talents, Liu Fang), National Natural Science Foundation of China(81600372) and China Endocrinology and Metabolism Talent Research Project (2021‐N‐03).

## CONFLICT OF INTEREST STATEMENT

The authors declare no competing interest.

## Data Availability

All data generated or analysed during this study are included in this published article. Data supporting the conclusions of this study are available from the authors. Further inquiries can be directed to the corresponding authors.
